# Supplementary material for: Electrical conductivity and magnetic dynamos in magma oceans of Super-Earths
Source: Nat Commun. 2018 Sep 24;9:3883. doi: 10.1038/s41467-018-06432-6 (PMC6155165; doi:10.1038/s41467-018-06432-6)
Supplement: Supplementary file 1 — Supplementary Information [file 41467_2018_6432_MOESM1_ESM.pdf]

Supplementary Information
Title: Electrical conductivity and magnetic dynamos in magma oceans of Super-Earths

François Soubiran and Burkhard Militzer

Supplementary Table I. Number of atoms and K-point grids used for the molecular dynamics and for the conductivity calculations. The n^3 grids are Monkhorst-Pack [1] grids.

Species	Phase	Number of formula unit	K-points MD	K-points conductivity
MgO	B1	32	2^3	4^3
	B2	64	2^3	2^3
	Liq.	32	Γ	4^3
	Liq.	60	Γ	4^3
$MgSiO_3$	ppv	12	Γ	4^3
	Liq.	12	Γ	2^3
SiO_2	pyr.	48	Γ	2^3
	Liq.	48	Γ	2^3

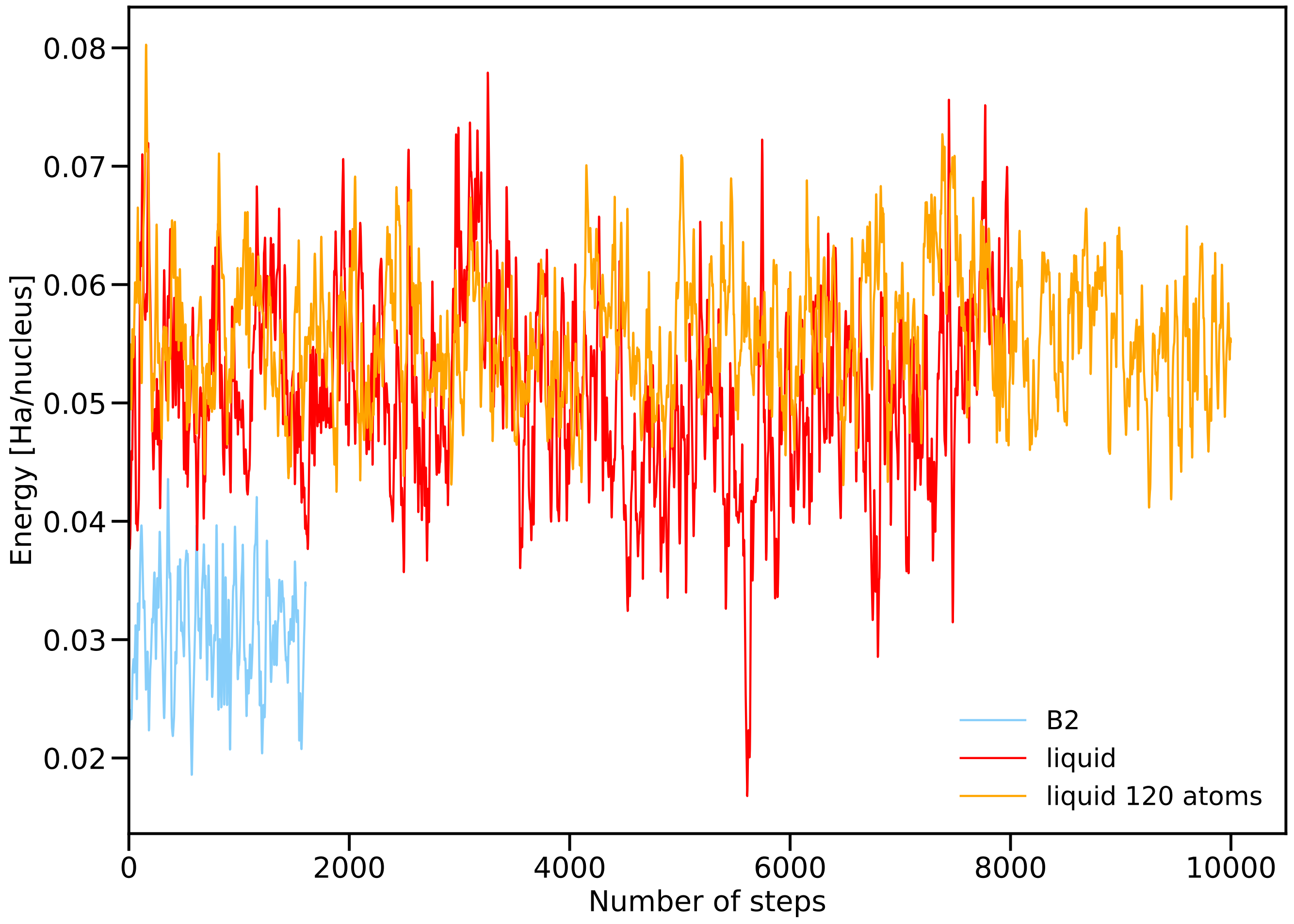

Supplementary Figure 1. Time evolution of the internal energy at equilibrium for a 64-atom and a 120-atom liquid MgO simulation and for a 128-atom B2 MgO simulation at around 470 GPa and 12000 K.

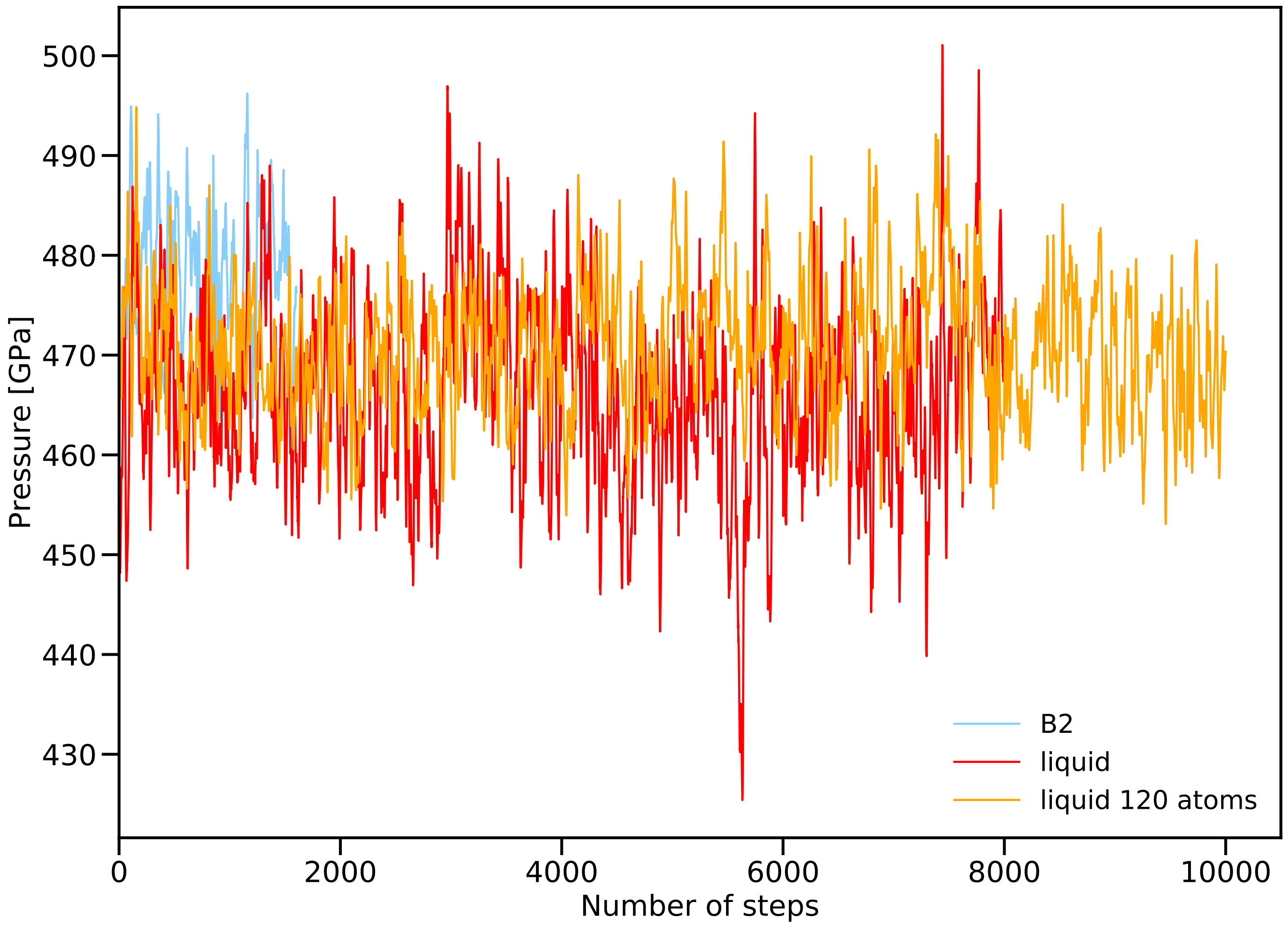

Supplementary Figure 2. Time evolution of the pressure at equilibrium for a 64-atom and a 120-atom liquid MgO simulation and for a 128-atom B2 MgO simulation at around 470 GPa and 12000 K.

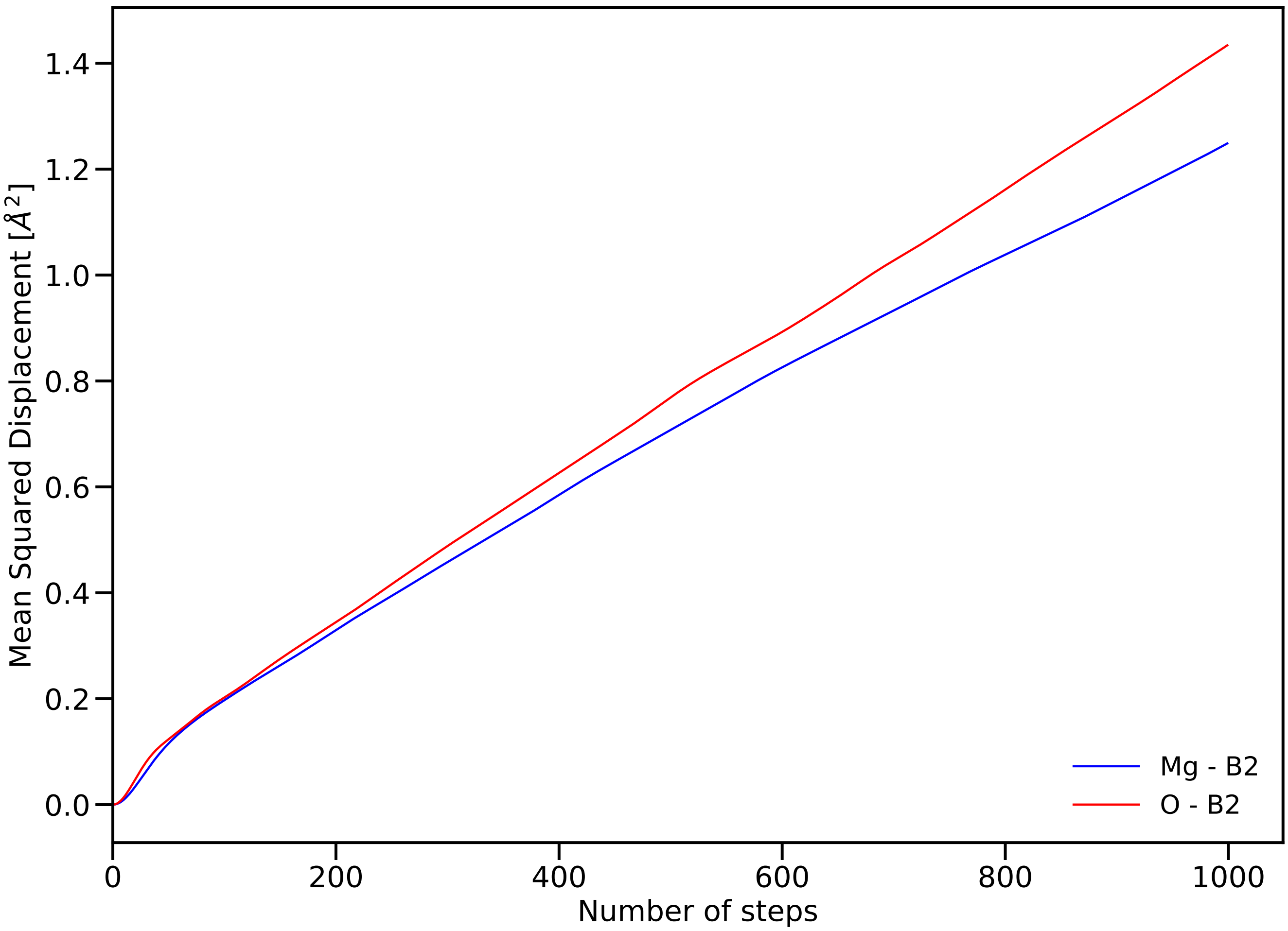

Supplementary Figure 3. Time evolution of the mean squared displacement of Mg and O nuclei in a liquid MgO simulation at around 470 GPa and 12000 K.

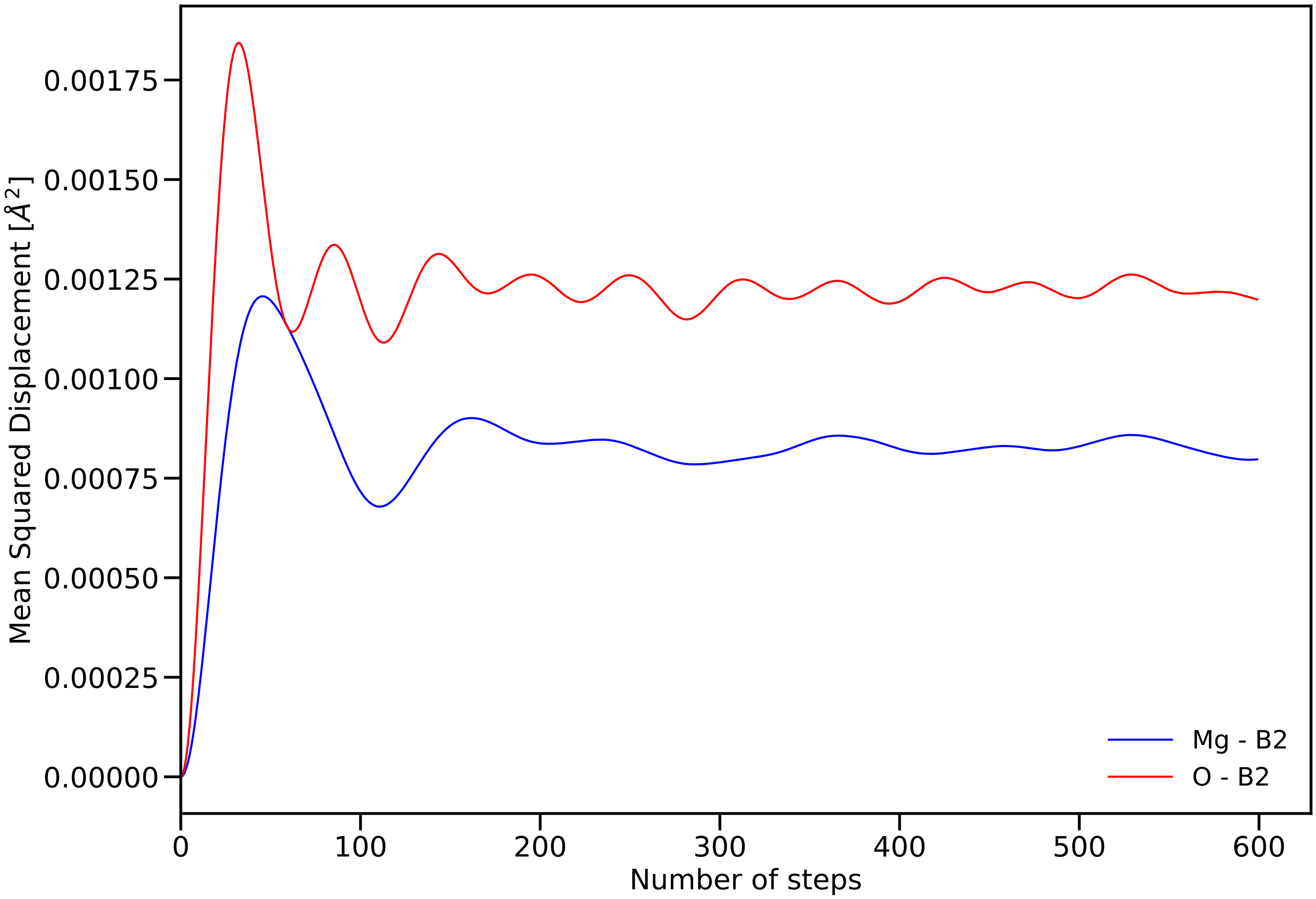

Supplementary Figure 4. Time evolution of the mean squared displacement of Mg and O nuclei in a solid B2 MgO simulation at around 470 GPa and 12000 K.

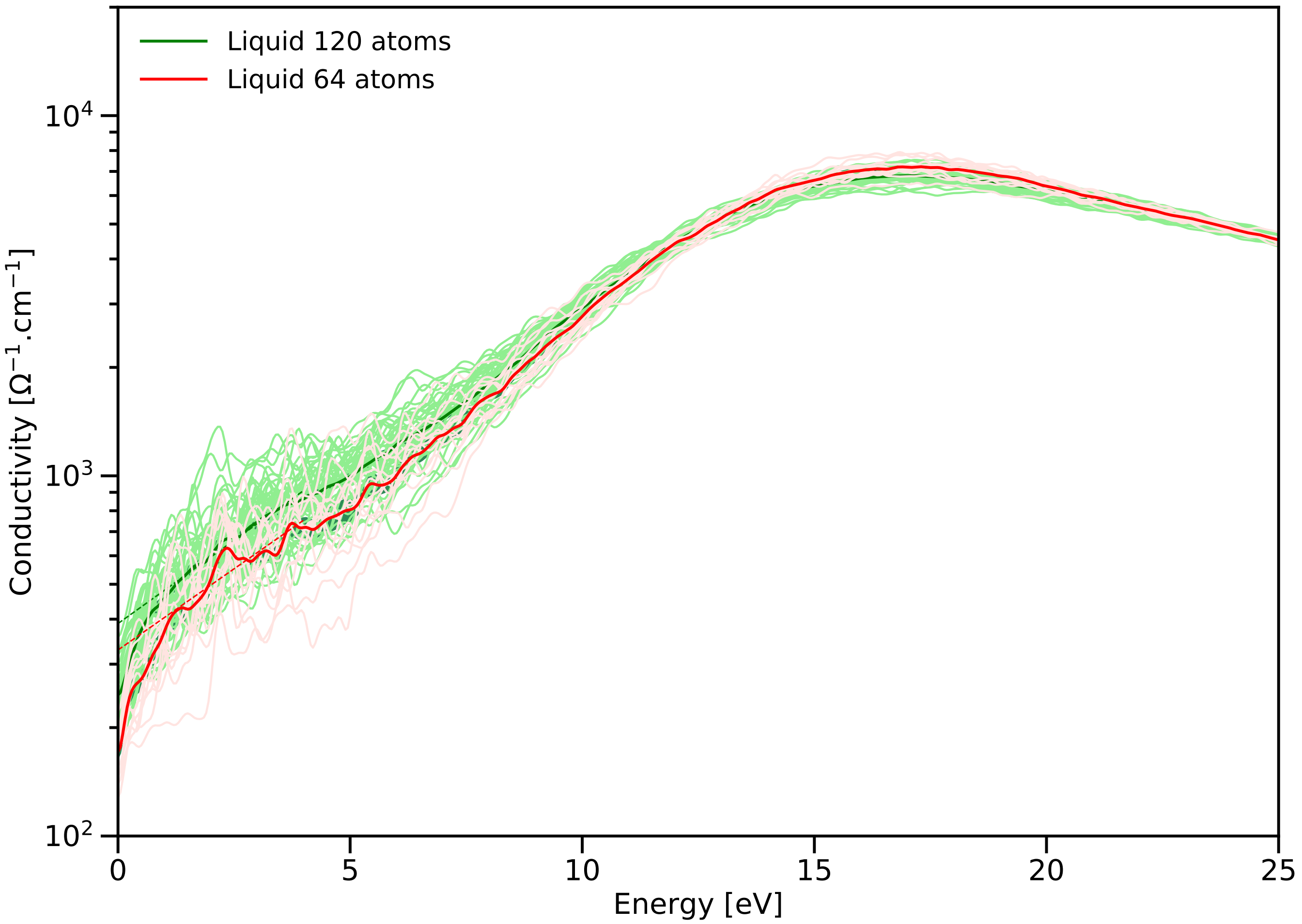

Supplementary Figure 5. Electric conductivity of liquid MgO at around 470 GPa and 12000 K, as a function of the excitation energy. The thin lines are the results of single snapshots and the thick lines are the average values for simulations with two different sizes. The dashed line is the extrapolation towards the low direct current value.

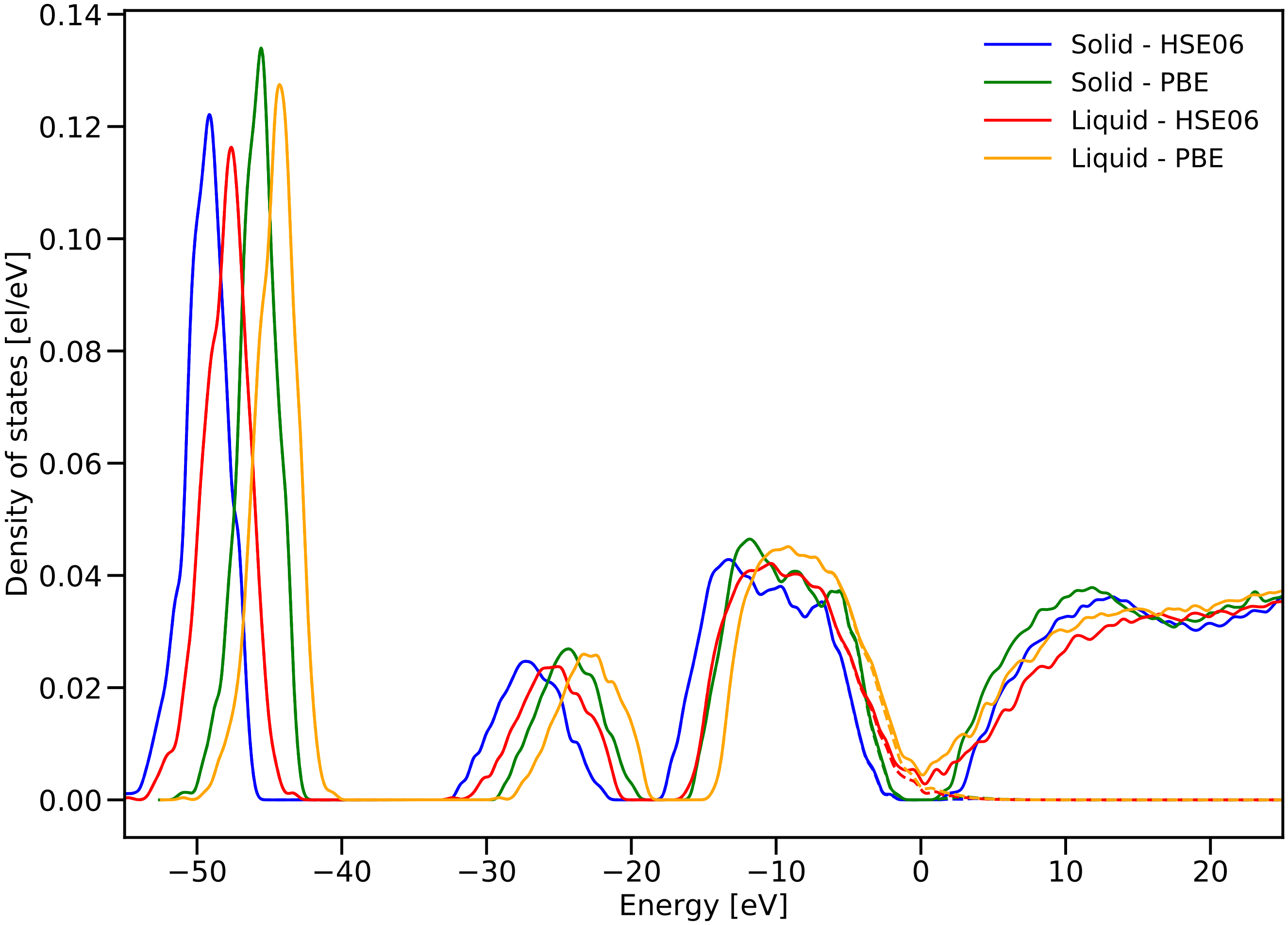

Supplementary Figure 6. DOS of liquid and B2 MgO at 470 GPa and 12000 K using PBE [2] and HSE06 [3, 4] on VASP.

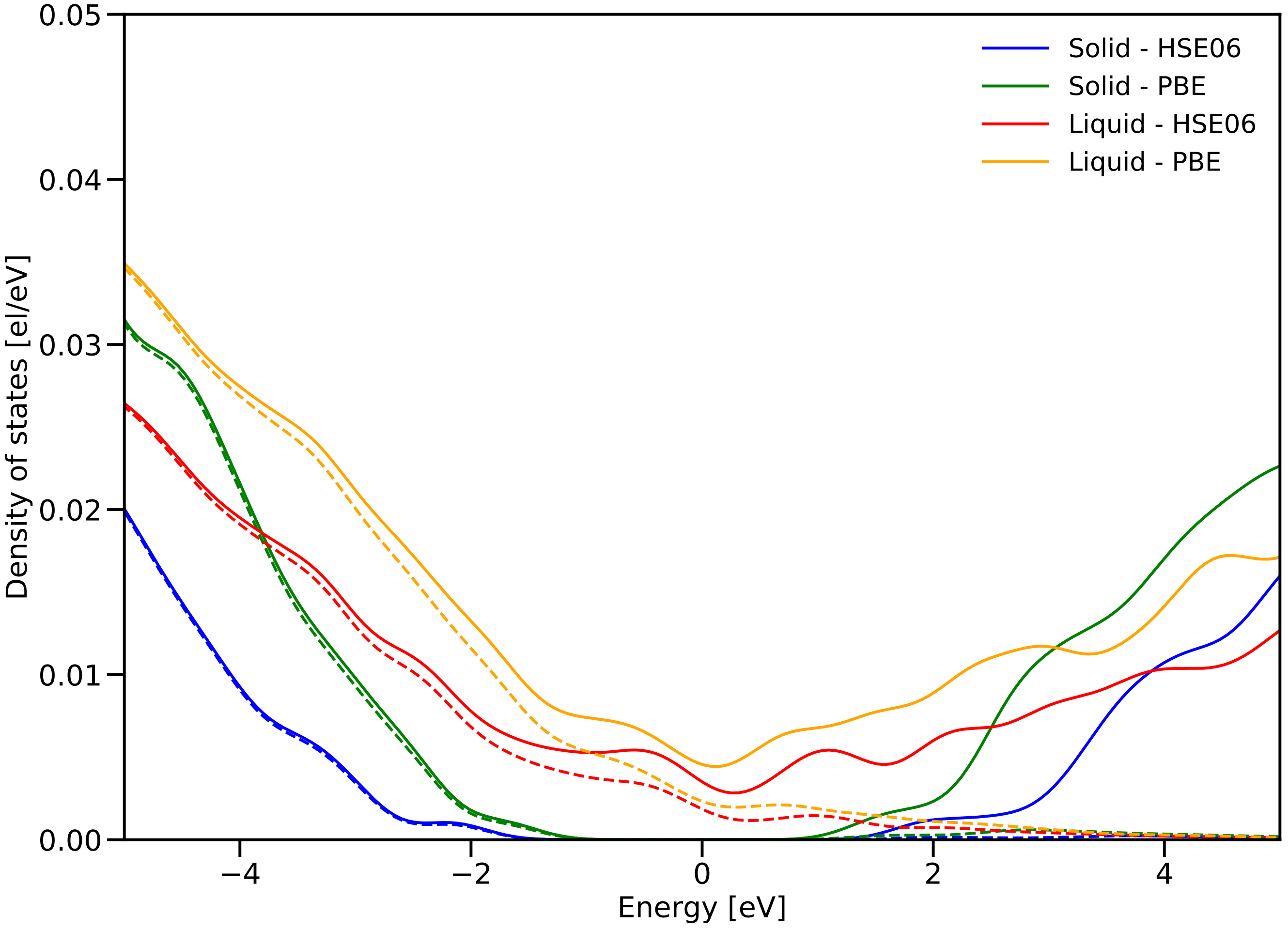

Supplementary Figure 7. Close in on the DOS in Fig. 6 around the Fermi level.

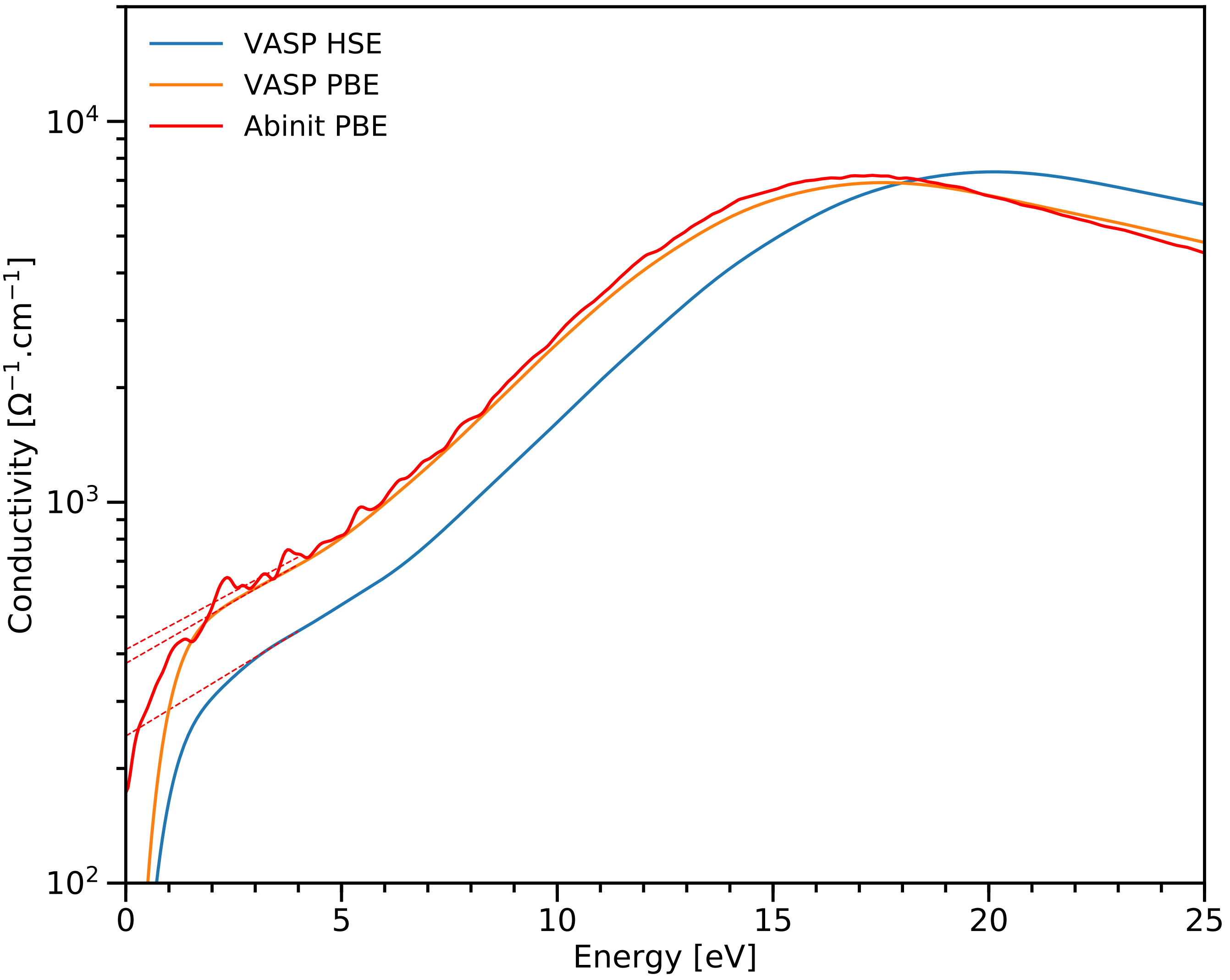

Supplementary Figure 8. Electrical conducitivity of liquid and B2 MgO at 470 GPa and 12000 K as a function of the excitation energy, using PBE [2] on VASP and Abinti, and HSE06 [3, 4] on VASP.

REFERENCES

1. Monkhorst, H. J. & Pack, J. D. Special points for Brillouin-zone integrations. *Phys. Rev. B* **13,** 5188 (1976).
2. Perdew, J. P., Burke, K. & Ernzerhof, M. Generalized Gradient Approximation Made Simple. *Phys. Rev. Lett.* **77,** 3865–3868 (1996).
3. Heyd, J., Scuseria, G. E. & Ernzerhof, M. Hybrid functionals based on a screened Coulomb potential Hybrid functionals based on a screened Coulomb potential. *J. Chem. Phys.* **118,** 8207 (2003).
4. Heyd, J., Scuseria, G. E. & Ernzerhof, M. Erratum: Hybrid functionals based on a screened Coulomb potential. *J. Chem. Phys.* **124,** 219906 (2006).
